# Blended Learning Compared With Face-to-Face Learning Among Family Medicine Residents: Randomized Controlled Trial

**DOI:** 10.2196/86387

**Published:** 2026-02-04

**Authors:** Pierre-Yves Meunier, Sophie Schlatter, Juliette Macabrey, Frédéric Zorzi, Thomas Colleony, Rémy Boussageon, Hubert Maisonneuve, Marion Lamort-Bouché

**Affiliations:** 1 Maison de santé pluriprofessionnelle et universitaire Beauvisage, Lyon, France; 2 Research on Healthcare Performance RESHAPE, Inserm U1290, Université Claude Bernard Lyon 1, Lyon, France; 3 Collège universitaire de médecine générale, Université Claude Bernard Lyon 1, 8 avenue Rockefeller, Lyon, 69003, France; 4 Healthcare Simulation Center, SIMULYON, Hospices Civils de Lyon, Université Claude Bernard Lyon 1, Lyon, France; 5 Maison de santé pluriprofessionnelle et universitaire Bel Air, Saint Priest, France; 6 Faculty of Education, University of Ottawa, Ottawa, ON, Canada; 7 French Military Medical School, Bron, France; 8 Laboratoire de biométrie et biologie évolutive UMR CNRS 5558, Université Claude Bernard Lyon 1, Université de Lyon, Lyon, France; 9 University Institute for Primary Care (IuMFE), Faculty of Medicine, University of Geneva, Geneva, Switzerland; 10 Mermoz Primary Health Center, Lyon, France

**Keywords:** primary health care, family practice, education, medical, graduate, internship and residency, distance, computer-assisted instruction, educational technology, self-assessment, educational measurement, randomized controlled trial

## Abstract

**Background:**

The medical education of French family medicine residents involves active, socioconstructivist-inspired small-group courses useful for skill acquisition. This is challenged by the increasing gap between the growing number of residents and the limited number of teachers. Blended courses have the potential to address this issue by reducing the duration of face-to-face sessions while preserving small-group courses.

**Objective:**

This study aimed to compare the effects of blended vs traditional, face-to-face, active, socioconstructivist learning on the acquisition of knowledge and skills by family medicine residents.

**Methods:**

We conducted a randomized controlled trial to compare a blended course and a traditional course. The blended course involved 2.5 hours of asynchronous e-learning and a 3-hour face-to-face session. The traditional course involved 5.5 hours of face-to-face teaching. Both courses were grounded in socioconstructivist principles and actively engaged residents. The primary outcome was residents’ self-assessment of knowledge and skills. Secondary outcomes included satisfaction with knowledge- or skill-related learning objectives and academic achievement at 6 months.

**Results:**

We included 155 family medicine residents (n=78, 50.3% in the blended course and n=77, 49.7% in the traditional course). There was no significant difference between groups regarding the primary outcome (mean difference 0.40 [maximum mean difference 20] points, 95% CI −0.21 to 1.02; *P*=.19; Cohen *d*=0.21). No significant differences were observed for the secondary outcomes except for knowledge self-assessment, which was higher in the blended course but not educationally meaningful (mean difference 0.40 [maximum possible 10] points, 95% CI 0.07-0.71; *P*=.02; Cohen *d*=0.39).

**Conclusions:**

Blended courses can help sustain socioconstructivist small-group teaching methods while accommodating a growing family medicine resident population, with no deleterious impact on knowledge and skill self-assessments.

## Introduction

The World Health Organization emphasizes the critical role of efficient primary care systems in ensuring population health [1]. However, many countries face a shortage of family physicians, which limits access to primary care services [2-4]. To address this issue, some countries, including France, have recently increased the number of medical students [5-7]. In France, family medicine residents are medical graduates enrolled in a 4-year family medicine residency program [8]. Their curriculum is competency-based, guided by a national framework [9]. The constructive alignment of competency-based educational objectives, learning activities, and assessment implies, among other things, the provision of active, socioconstructivist-inspired courses [10,11]. Social constructivism emphasizes the role of social interactions and collaboration for effective learning [12]. As a result, residents engage in a variety of collaborative learning activities with 3 to 25 participants per group. Reports of these activities, individual narrative medicine work, and assessments are collected in a portfolio. However, from the perspective of health profession educators, it is difficult to maintain feasibility with increasing class sizes [13]. This difficulty is particularly acute for family medicine residents because their growing number has outpaced the availability of teaching hours, threatening the quality of their education.

Face-to-face traditional learning relies on nondigital materials and human interaction [14]. Blended learning (BL) combines face-to-face education with asynchronous or synchronous e-learning [15]. BL is often used to shift from a teacher-centered, behaviorist instructional model to a student-centered, socioconstructivist instructional model [16]. In blended courses, face-to-face sessions reinforce knowledge and develop skills through the collective mobilization of knowledge acquired during e-learning [17]. Two meta-analyses found that BL had a more positive effect on knowledge acquisition compared to traditional education in health professions (including medical students) but with large heterogeneity in the results [18,19]. This heterogeneity can be explained by differences in student populations and missing details about the instructional approach. Moreover, these studies did not evaluate the effect of BL on skill acquisition. While BL appears to improve satisfaction among both students and teachers, no meta-analysis has specifically evaluated this outcome. Teachers have reported that BL has the potential to increase interactions with students [20] and allow educators to develop more content [21] and they felt greater job satisfaction with this modality [22]. Students have generally been satisfied [23].

While existing studies suggest that BL is generally effective, its effectiveness compared with an already active, socioconstructivist, face-to-face course remains unexplored. Moreover, its effect on skill acquisition among postgraduate medical trainees (residents) remains underexplored. In this study, we compared the effects of a blended course (asynchronous active e-learning with shorter socioconstructivist face-to-face sessions) vs a fully face-to-face socioconstructivist course on the acquisition of knowledge and skills by family medicine residents. While implementing BL could mitigate the shortage of teachers by shortening face-to-face sessions, reduced in-person interaction may limit feedback and engagement, potentially leading to lower knowledge and skill acquisition.

## Methods

### Study Design

We conducted a single-blind randomized controlled trial with 2 parallel arms. The experimental arm received a blended course comprising an asynchronous e-learning module (2.5 hours; 45% of the course) followed by a face-to-face session (3 hours; 55% of the course). The control arm received the usual face-to-face traditional course (5.5 hours; 100% of the course). The trial was conducted between May 2024 and November 2024 at Université Claude Bernard Lyon 1 (France). It followed the CONSORT (Consolidated Standards of Reporting Trials) guidelines [24] and was prospectively registered on ClinicalTrials.gov (NCT06409273).

### Sample and Setting

All first-year family medicine residents at Université Claude Bernard Lyon 1 were administratively enrolled, with no exclusion criteria. The course was delivered over a single day. Residents were assigned to 1 of 2 available dates. Participants were told that the study compared different teaching methods, with no further detail. They were block randomized using random sequences of block sizes of 4 and 8 in a 1:1 allocation ratio. Randomization was performed by the first author using random permuted blocks generated online [25]. On each teaching date, 4 groups were formed (2 per arm), with a maximum of 23 participants per group. Classrooms were configured similarly, with tables arranged in a U shape and spaces reserved for breakout work.

The 5 teachers were randomized via coin toss to 1 of the 2 study arms. Teachers who taught on both dates crossed over to the other arm on the second day. For each arm, we developed detailed, time-stamped scripts to ensure instructional fidelity and equivalence of content and duration.

### Educational Intervention and Conceptual Framework

The course prepared residents to complete the thesis required for full qualification in France. This is a professionally oriented doctoral thesis publicly defended before an examination board. It is required for the award of the national Doctor of Medicine degree, which permits registration with the French Medical Council for independent practice. Distinct from a PhD, it is a concise, supervised scholarly work demonstrating the candidate’s ability to design, conduct, and communicate research relevant to medical practice. We specified 5 process-oriented learning objectives: 3 for knowledge acquisition and 2 for skill development (Table 1).

**Table 1.** Learning objectives across study arms.

Learning objective	Study arm	
	Blended course	Traditional course
Knowledge oriented		
Identify the practical organization and regulatory requirements of the medical thesis	e-Learning	Face-to-face
Understand the different steps of a research project	e-Learning	Face-to-face
Summarize the medical thesis application form requirements	e-Learning	Face-to-face
Skill oriented		
Frame the research question for a thesis project	Face-to-face	Face-to-face
Assess the feasibility of a medical thesis project	Face-to-face	Face-to-face

The blended course sequenced active knowledge construction online with supervised skill development in person. We made the alignment between the 2 sessions explicit to residents to underscore the coherence between the learning objectives and the learning methods. The blended course arm began with an e-learning session grounded in self-regulated learning (SRL). SRL has been defined as self-generated thoughts, feelings, and actions that are planned and cyclically adapted to the attainment of personal goals [26]. Process-oriented learning objectives (Table 1) for knowledge acquisition were designed to activate the SRL forethought phase (goal setting and strategy selection) [27]. We aimed to help residents identify immediate next steps (securing a supervisor, conducting a literature review, and framing a research question), whereas strategy selection focused on learning how to choose an appropriate research method. Formative checkpoints and immediate feedback were embedded through interactive HTML5 Package activities—videos with decision points (“crossroads”), multiple-choice questions, drag-the-words items, and navigation hot spots. This design supported ongoing self-monitoring and adaptive control (comparison to standards and adjustment), thereby reducing discrepancies between current understanding and target performance. The accompanying explanatory text and curated external links to websites or documents enabled resource management and self-paced elaboration, further supporting SRL. The e-learning module was implemented online in the course management system Moodle (Moodle HQ; Multimedia Appendix 1).

The traditional course arm began with the collaborative construction of a knowledge base anchored in the knowledge-oriented learning objectives. Teachers acted as facilitators, providing key information and externalizing group thinking in mind maps that made success criteria and progress visible for monitoring.

Skill-oriented learning objectives were addressed identically in both arms during a face-to-face session. For the socioconstructivist activities, peer evaluation of thesis projects was organized in breakout groups, which discussed the research question and feasibility. These activities provided external feedback according to standards explicitly set by teachers, closing the control loop and supporting the SRL reflection phase (self-evaluation and calibration). To stimulate motivational processes and goal setting, we highlighted the timeline leading up to the thesis [28].

### Data Collection and Outcomes

Data were collected at the end of the teaching day via individualized links to an online form (Multimedia Appendix 2). Participants provided demographics (gender, age, research experience, and progress stage of the thesis project before teaching) and completed two 7-point Likert questionnaires: self-assessed learning (3‐8 items per objective) and satisfaction (1 item per objective). To ensure content validity and constructive alignment, all items were copied verbatim from prespecified intended learning outcomes (Multimedia Appendix 3), yielding a criterion-referenced measure. Item order was randomized.

The primary outcome was a composite score of 20 points reflecting residents’ self-assessment of knowledge and skills. It was the sum of 2 secondary outcomes: a knowledge self-assessment score (10 points) and a skill self-assessment score (10 points).

The secondary outcomes were as follows:

1. Overall satisfaction (20 points); sum of secondary outcomes 4 and 5
2. Self-assessment of knowledge acquisition (10 points), with equal weight given to each knowledge-oriented learning objective
3. Self-assessment of skill acquisition (10 points), with equal weight given to each skill-oriented learning objective
4. Satisfaction with knowledge-oriented learning objectives (10 points)
5. Satisfaction with skill-oriented learning objectives (10 points)
6. Academic achievement (submission of a thesis proposal form at 6 months; yes or no)

### Statistical Analysis

Analysis was performed using R (version 4.4.3; R Foundation for Statistical Computing) in RStudio (version 2024.12.1; Posit PBC) [29]. Because the primary outcome was assessed only at the end of the intervention, participants with missing outcome data were excluded from the analysis, resulting in a modified intention-to-treat approach with a complete-case analysis. Missing outcomes were not imputed, and reasons for missing data were documented. Multiplicity across secondary outcomes was controlled using a hierarchical fixed-sequence procedure that followed the prespecified order listed above (1 to 6). Each hypothesis was tested 2 sided at α=.05, and testing stopped at the first nonsignificant result (subsequent analyses were exploratory).

We modeled the primary outcome using multivariable linear regression, estimating the arm effect (blended vs traditional) while adjusting for a prespecified set of the following predictor variables. Precourse framing of a research question and prior research experience were included as proxies for baseline research preparedness. Full completion of another Moodle e-learning course was included as a proxy for familiarity with digital learning environments. Unblinding was included to account for potential expectancy or performance effects linked to perceived allocation. Teacher and course day were included to account for contextual instructional and organizational variability. Age and gender were included as standard demographic covariates. Model assumptions were checked (Multimedia Appendix 4). We report β coefficients with 95% CIs and adjusted *R*^2^. For power, a sample size of 144 (72 per arm) was planned to provide 85% power to detect a 2-point difference on a 20-point scale (SD 4; 2-sided α=.05). The SD was conservatively inflated given limited prior data. Internal consistency per objective was estimated using the Cronbach α.

### Ethical Considerations

This trial received ethics approval from the Comité d’Ethique de la Recherche du Collège Universitaire de Médecine Générale (CUMG-IRB 2024-04-30-02). Participants received oral and written information about the study and provided written informed consent. The data collected complied with the European Union’s General Data Protection Regulation and with the data management policy of Université Claude Bernard Lyon 1 as confirmed by the university’s data protection officer. No compensation was provided to participants.

## Results

### Overview

**Figure 1.**
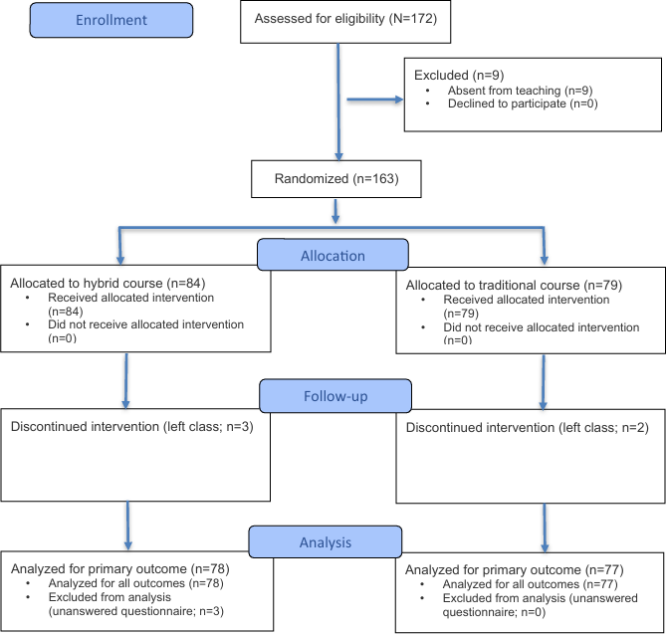
CONSORT (Consolidated Standards of Reporting Trials) flow diagram.

We included 163 family medicine residents randomized to the blended (n=84, 51.5%) and the traditional (n=79, 48.5%) course. A total of 155 participants completed the online form at the end of the day: 78 (50.3%) in the blended course, and 77 (49.7%) in the traditional course (Figure 1). Table 2 presents the participants’ characteristics. The mean age of the participants was 25.7 (SD 3.54) years, and most (112/155, 72.3%) were women. Most participants (114/155, 73.5%) had an idea for their thesis topic. A minority had framed their research question (24/155, 15.5%) or already had a thesis supervisor (22/155, 14.2%). In total, 29.7% (46/155) of the participants reported having research experience, and 3.9% (6/155) had completed a full master’s degree or a PhD in addition to their medical studies. Less than a quarter of the participants (32/155, 20.6%) had already completed a full e-learning course on the university’s digital learning platform (Moodle). Nearly a third of the participants (48/155, 31.0%) were unblinded at the end of the course. Five teachers offered the course, and 3 of them taught in both arms.

**Table 2.** Baseline participant demographics.

	Blended course (n=78)	Traditional course (n=77)
Age (y), mean (SD)	25.8 (4.1)	25.6 (2.8)
Gender, n (%)		
Men	19 (24.4)	22 (28.6)
Women	58 (74.4)	54 (70.1)
Nonbinary	1 (1.3)	1 (1.3)
Research experience (master’s or PhD), n (%)		
None	45 (57.7)	57 (74.0)
Master’s—first year	28 (35.9)	18 (23.4)
Master’s—full	4 (5.1)	1 (1.3)
PhD	1 (1.3)	0 (0.0)
Achieved before the course, n (%)		
Idea for thesis subject	62 (79.5)	52 (67.5)
Research question formulated	10 (12.8)	14 (18.2)
Found a thesis supervisor	10 (12.8)	12 (15.6)
Other e-learning course completed on Moodle	21 (26.9)	11 (14.3)
Unblinded^a^	23 (29.5)	25 (32.5)

a Aware of group allocation.

The proportion of postrandomization exclusions was low (8/163, 4.9%) and similar in both group allocations (Multimedia Appendix 5). It was due to missing outcome data, mostly because of work-related early departure.

### Primary Outcome

Knowledge and skill self-assessment did not differ significantly between the blended and traditional courses (mean difference 0.40 [maximum possible 20] points, 95% CI −0.21 to 1.02; *P*=.19; Cohen *d*=0.21; Table 3). This result remained not statistically significant after adjustment (Table 4). The most significant predictor of the primary outcome was having formulated a research question before the course, with a β coefficient of 1.87 (95% CI 1.06-2.68; *P*<.001). Unblinding during the day and teacher identity were not significantly associated with the primary outcome (*P*=.13).

**Table 3.** Education outcomes.

Outcome measure (KP^a^ level)	Blended course (n=78)	Traditional course (n=77)	Mean difference (95% CI)	Cohen *d*	*P* value
Self-assessment of knowledge and skills (20 points; KP level: 2), mean (SD)	14.66 (1.92)	14.26 (1.94)	0.4 (–0.21 to 1.01)	0.21	.19^b^
Overall satisfaction (20 points; KP level: 1), mean (SD)	14.93 (2.80)	14.77 (2.55)	0.16 (–0.68 to 1.01)	0.06	.70^b^^,c^
Self-assessment of knowledge acquisition (10 points; KP level: 2), mean (SD)	7.49 (0.98)	7.09 (1.04)	0.4 (0.07 to 0.71)	0.38	.01^b^^,^^c^
Self-assessment of skill acquisition (10 points; KP level: 2), mean (SD)	7.18 (1.20)	7.17 (1.17)	0.01 (–0.27 to 0.27)	0.009	.76^c^^,^^d^
Satisfaction with knowledge-oriented educational objectives (10 points; KP level: 1), mean (SD)	7.54 (1.53)	7.42 (1.30)	0.12 (–0.00005 to 0.55)	0.08	.34^c^
Satisfaction with skill-oriented educational objectives (10 points; KP level: 1), mean (SD)	7.40 (1.48)	7.36 (1.46)	0.04 (–0.00007 to 0.83)	0.03	.64^c^^,^^e^
Submission of a thesis application form at 6 months (KP level: 3), n (%)	11 (14.1)	10 (13.0)	1.09 (0.39 to 3.1)^f^	—^g^	>.99^d^

a KP: Kirkpatrick [30].

b Student *t* test (2 sided).

c Once a hypothesis was tested and found not to be significantly different from the null hypothesis, all subsequent tests and results were considered exploratory. The secondary objectives described earlier were ranked according to this procedure.

d Fisher exact test (2-sided).

e Mann-Whitney *U* test.

f Odds ratio reported for this value.

g Not applicable.

**Table 4.** Effect of the study arm (blended or traditional course) on the primary outcome while adjusting for prespecified predictor variables.^a^

Predictor variable	β^b^ (95% CI)	*P* value
Research question framed before the course (yes)	1.87 (1.06 to 2.68)	<.001
Research experience (yes)	−0.27 (–0.92 to 0.37)	.40
Unblinding (yes)	−0.52 (–1.20 to 0.16)	.13
Teacher 1	0.69 (–0.48 to 1.87)	.25
Teacher 2	0.91 (0.06 to 1.87)	.04
Teacher 3	−0.13 (–1.33 to 1.05)	.82
Teacher 4	−0.16 (–1.01 to 0.69)	.71
Another e-learning course completed on Moodle (yes)	0.09 (–0.63 to 0.82)	.79
Age	−0.08 (–0.16 to 0.01)	.07
Gender—man	0.26 (–0.40 to 0.93)	.43
Gender—nonbinary	1.41 (–1.20 to 4.03)	.28
Course day (day 2)	0.02 (–0.72 to 0.76)	.94
Arm (traditional course)	−0.23 (–0.99 to 0.53)	.55

a After adjustment for prespecified predictor variables, there were no significant differences between the blended and traditional courses regarding the primary outcome (*P*=.55). Given the exploratory nature of the variable selection procedure, adjusted estimates should be interpreted cautiously. *R*2 is the proportion of the variance explained by the model (multiple *R*2=0.21; adjusted *R*2=0.13; *P*=.001 for the model).

b Coefficient representing the expected change in the primary outcome per 1-unit increase in the predictor variable.

### Secondary Outcomes

As an exploratory result, the knowledge self-assessment was significantly higher in the blended course (mean difference 0.40 [maximum possible 10], 95% CI 0.07-0.71; *P*=.01; Cohen *d*=0.38).

The skill self-assessment by residents was not significantly different between the traditional and blended courses (*P*=.76). Their overall satisfaction or specific satisfaction regarding knowledge- or skill-oriented learning objectives did not differ between the traditional and blended courses. The submission of a thesis proposal form was also not found to be different between the 2 groups (11/78, 14.1% in the BL arm and 10/77, 13.0% in the traditional course arm).

### Internal Consistency

Items assessing each educational objective showed acceptable Cronbach α values (>0.70) for all but one educational objective, which exhibited lower internal consistency (Cronbach α=0.47; Multimedia Appendix 6).

## Discussion

### Principal Findings

Among French family medicine residents, self-assessed knowledge and skills did not differ between the blended and traditional courses. The between-arm mean difference was small, and the 95% CI was narrow, indicating low uncertainty around the estimated difference. At 6 months, academic achievement (thesis proposal submission) did not differ between arms. The only statistically significant finding—an exploratory, slightly higher knowledge score in the blended arm—was not educationally meaningful.

### Comparison With Other Studies

To our knowledge, no randomized controlled trial has evaluated whether partially replacing an already active, socioconstructivist face-to-face course with a blended format affects residents’ self-reported knowledge and skills. A mixed methods randomized controlled trial compared a blended evidence-based medicine course with a lecture-based (behavioral) traditional course and found no significant difference in objectively measured knowledge and skills. However, the blended group reported higher self-efficacy, attitudes, and self-reported behaviors. Focus groups suggested a clear learner preference for the blended, integrated approach [31]. In health profession education, a meta-analysis comparing flipped classrooms with traditional, lecture-based (behavioral) courses found modest improvements in learning [32]. Flipped classrooms are a common BL configuration comparable to our study intervention combining preclass online preparation with active in-class work. Other observational studies have demonstrated the relevance of BL in medical education, most notably since the COVID-19 pandemic [33,34]. A recent meta-analysis of systematic reviews recommended further research to determine the relative benefits of BL in each individual context. It also confirmed our exploratory finding of improved knowledge acquisition with BL [35]. Moreover, a randomized controlled trial with undergraduate medical students demonstrated the noninferiority of BL vs traditional education in technical skill performance (life-saving trauma skills) and retention [36]. A randomized controlled trial of postgraduate medical students was conducted to compare BL to traditional education, and it showed significantly greater efficacy of BL for developing defibrillator technical skills [37]. Our trial extends the evidence to academic skills related to developing a research project (thesis); it suggests that shortening face-to-face time through a structured e-learning component can yield comparable educational outcomes when the face-to-face component remains active and socioconstructivist.

More broadly, syntheses of digital education in the health professions consistently report large benefits vs no intervention [38] but little or no differences vs nondigital instruction [39].

### Implications

This study may reassure teaching teams that BL can produce short-term educational outcomes comparable to those of traditional active and socioconstructivist education among residents, particularly in settings with constrained teaching capacity. Still, the implementation of BL does not address all the issues associated with the growing disparity between the number of teachers and residents. First, BL may not always be appropriate, either because of a misalignment between educational objectives aimed at developing complex competencies and the learning modality or because of the substantial resources required to develop online courses that effectively address such competencies [40]. For example, a course focused on professional communication competencies might be better suited to face-to-face relational simulation as the sole learning modality. Second, monitoring residents’ acquisition of generic knowledge, skills, and competencies based on a national framework still necessitates frequent, individualized human contact. Third, strategies facilitating students’ SRL may imply their supervision at the asynchronous e-learning phase, which would not allow for a significant reduction in teaching time.

The transition from traditional to blended education represents a substantial organizational shift. Teachers must allocate time to review asynchronous assessments and prepare collective feedback for the face-to-face session. It is important to estimate the time required for residents to complete all e-learning modules to avoid unintentionally increased workload. Strategies to promote SRL are also needed to optimize educational outcomes [41,42]. For example, providing dedicated time and space at the university to complete the e-learning module may help residents allocate time for it. Furthermore, working independently at home may increase feelings of stress and anxiety, as was observed during the COVID-19 pandemic [43].

### Limitations and Strengths

One limitation of this study was the reliance on self-assessment rather than objective measures. Incorporating objective performance measures would enhance validity and mitigate self-report bias, for example, through standardized pretest-posttest knowledge tests and rubric-based scoring of residents’ performance on key skills (framing a research question and assessing feasibility) rated by trained assessors. Still, formative self-assessment is integral to self-regulation, enabling feedback on progress relative to intended learning outcomes [44,45]. Moreover, the e-learning module was an initial version. A well-established, iteratively refined module might have produced higher scores among residents randomized to the BL arm. The e-learning module was completed immediately before the face-to-face session, which constrained residents’ autonomy to plan their study and is partly misaligned with SRL. This design ensured full completion of the e-learning module, reduced the risk of early unblinding or contamination between groups before the face-to-face session, and standardized learning conditions (same setting and time on task). However, it did not capture real-life environmental variability, and the immediate sequencing in BL may also have facilitated knowledge activation for the subsequent face-to-face session. Although a high proportion of participants were unblinded by the end of the teaching day—a potential limitation—this variable was not associated with the primary outcome in multivariate analysis. The complete-case analysis may have introduced attrition bias; however, the proportion of missing outcome data remained low (8/163, 4.9%) and similar in both group allocations.

Regarding strengths, the narrow 95% CI suggests that an educationally meaningful difference of ≥10% (≥2/20 points) between groups would have been detected even though the study was not designed to test equivalence or noninferiority. A formal noninferiority trial is still needed. Furthermore, the lack of significant teacher effects on the primary outcome indicates that the standardized, timed script effectively minimized instructor-specific variability.

### Conclusions

Self-assessment of knowledge and skills by family practice residents did not differ between blended and traditional courses. This study can reassure educators that BL may produce short-term educational outcomes (knowledge and academic skills) comparable to those of traditional active and socioconstructivist education among residents, particularly in settings with constrained teaching capacity.

## Supplementary material

10.2196/86387 Multimedia Appendix 1 Screenshots of the e-learning module.

10.2196/86387 Multimedia Appendix 2 Data collection form.

10.2196/86387 Multimedia Appendix 3 Questionnaire items for self-assessment and satisfaction (verbatim wording from intended learning outcomes).

10.2196/86387 Multimedia Appendix 4 Multiple linear regression model: checking assumptions.

10.2196/86387 Multimedia Appendix 5 Reasons for postrandomization exclusion in complete-case analysis.

10.2196/86387 Multimedia Appendix 6 Internal consistency of family medicine residents’ self-assessment: Cronbach α values for each educational objective.

10.2196/86387 Checklist 1 CONSORT checklist.

## Abbreviations

BL: blended learning
CONSORT: Consolidated Standards of Reporting Trials
SRL: self-regulated learning
