# Supplementary figures and images for: Blended Learning Compared With Face-to-Face Learning Among Family Medicine Residents: Randomized Controlled Trial
Source: JMIR Med Educ. 2026 Feb 4;12:e86387. doi: 10.2196/86387 (PMC12871943; doi:10.2196/86387)

**Multimedia Appendix 1. Visuals of the e-learning module**

**
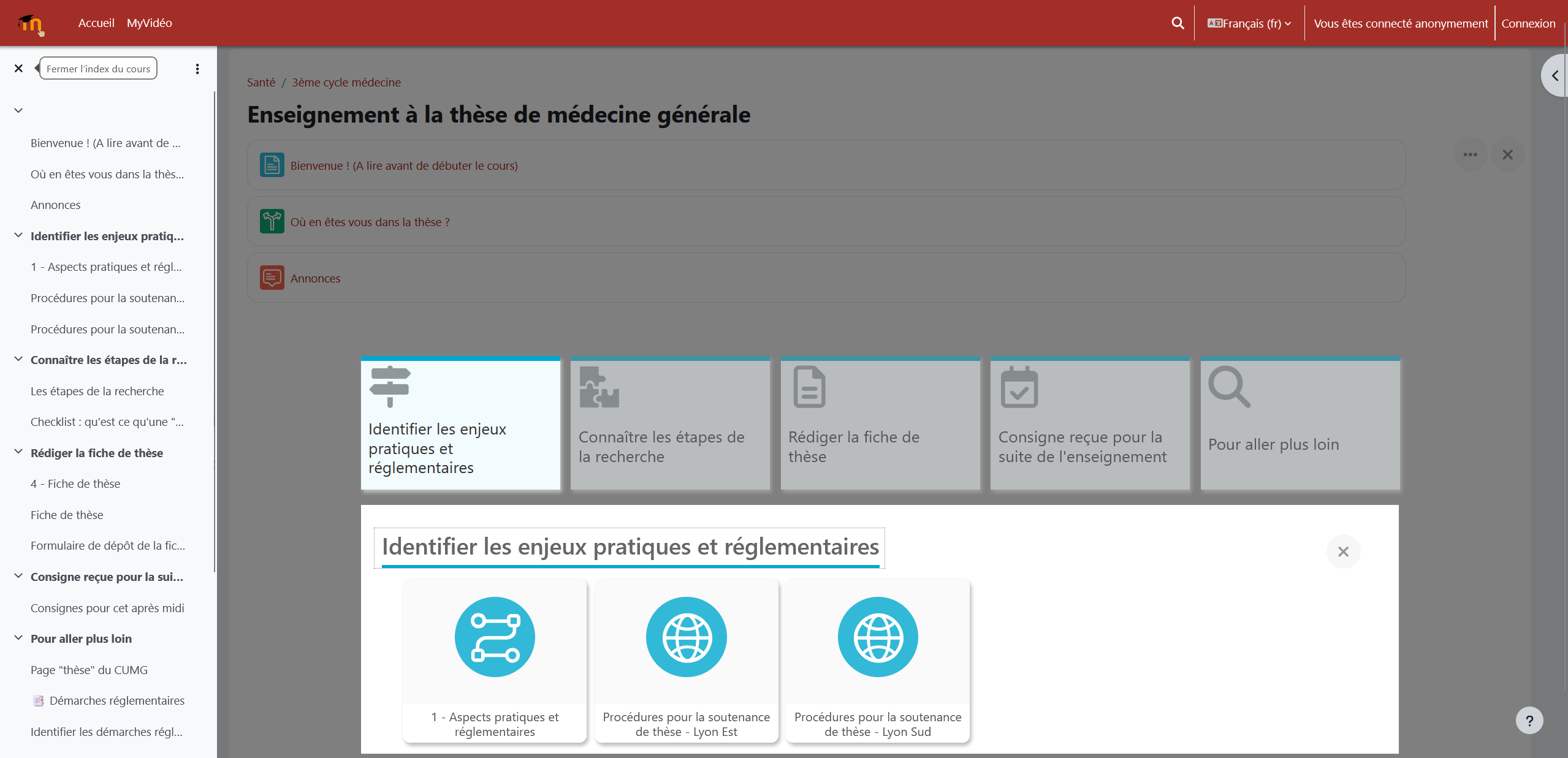
**

**
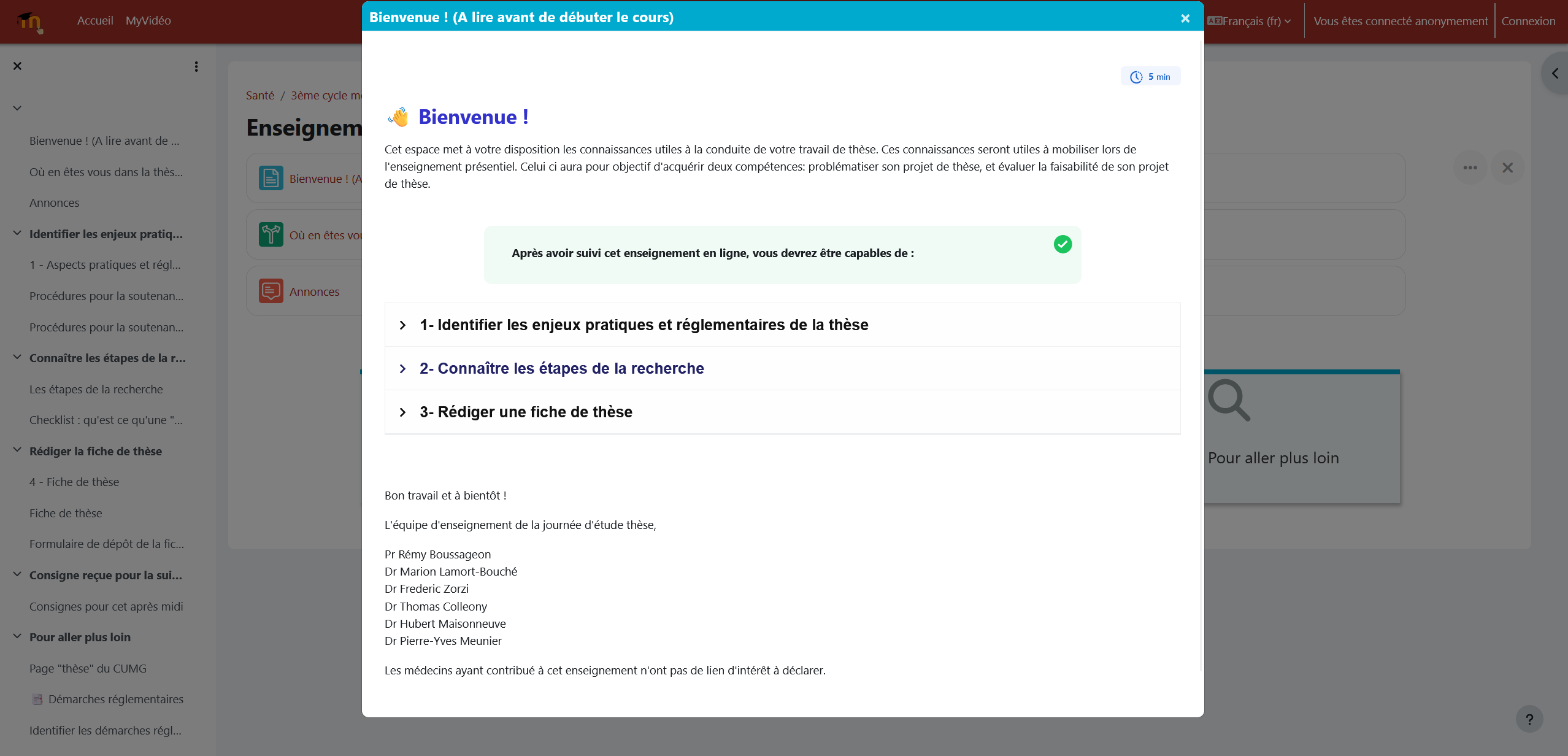
**

**
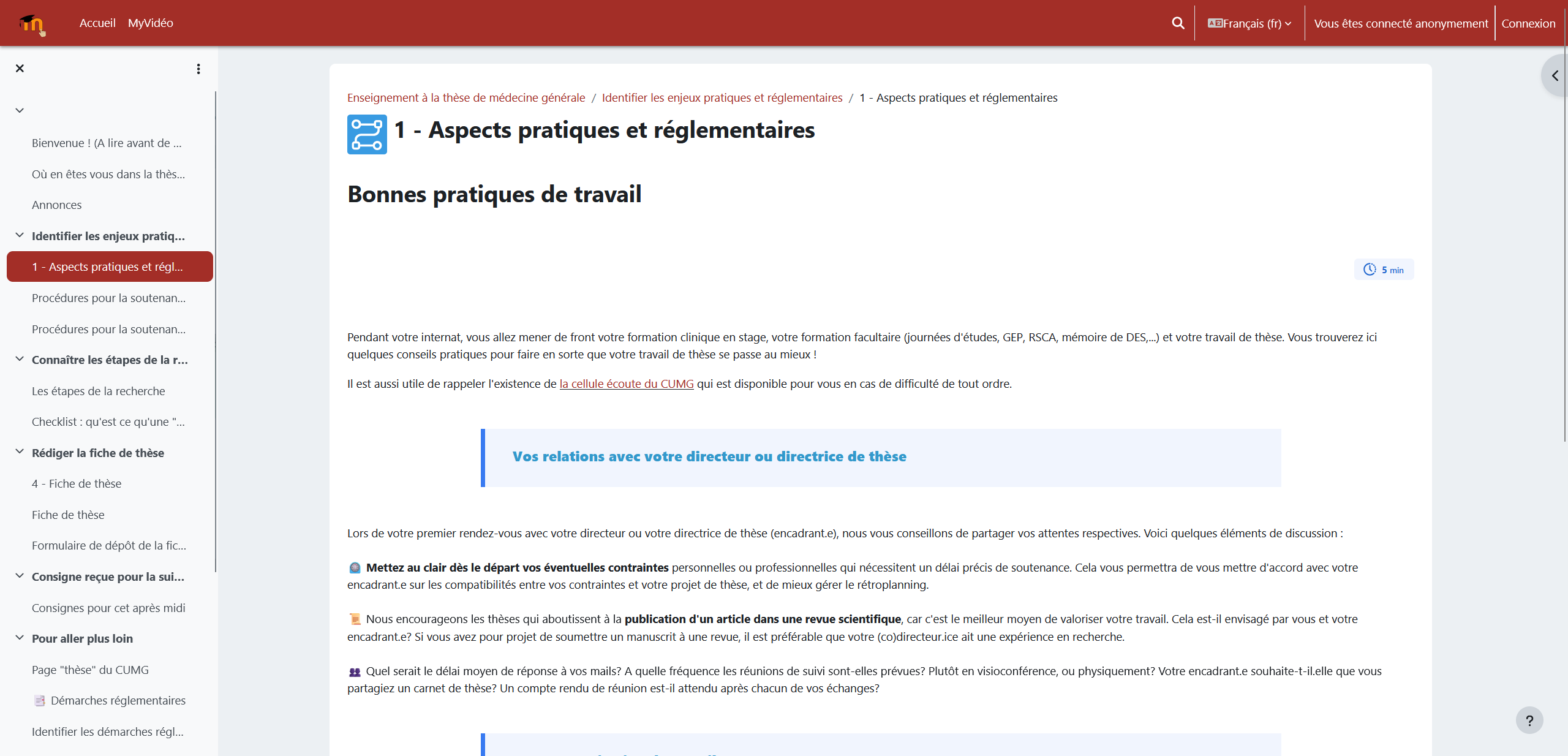
**

**
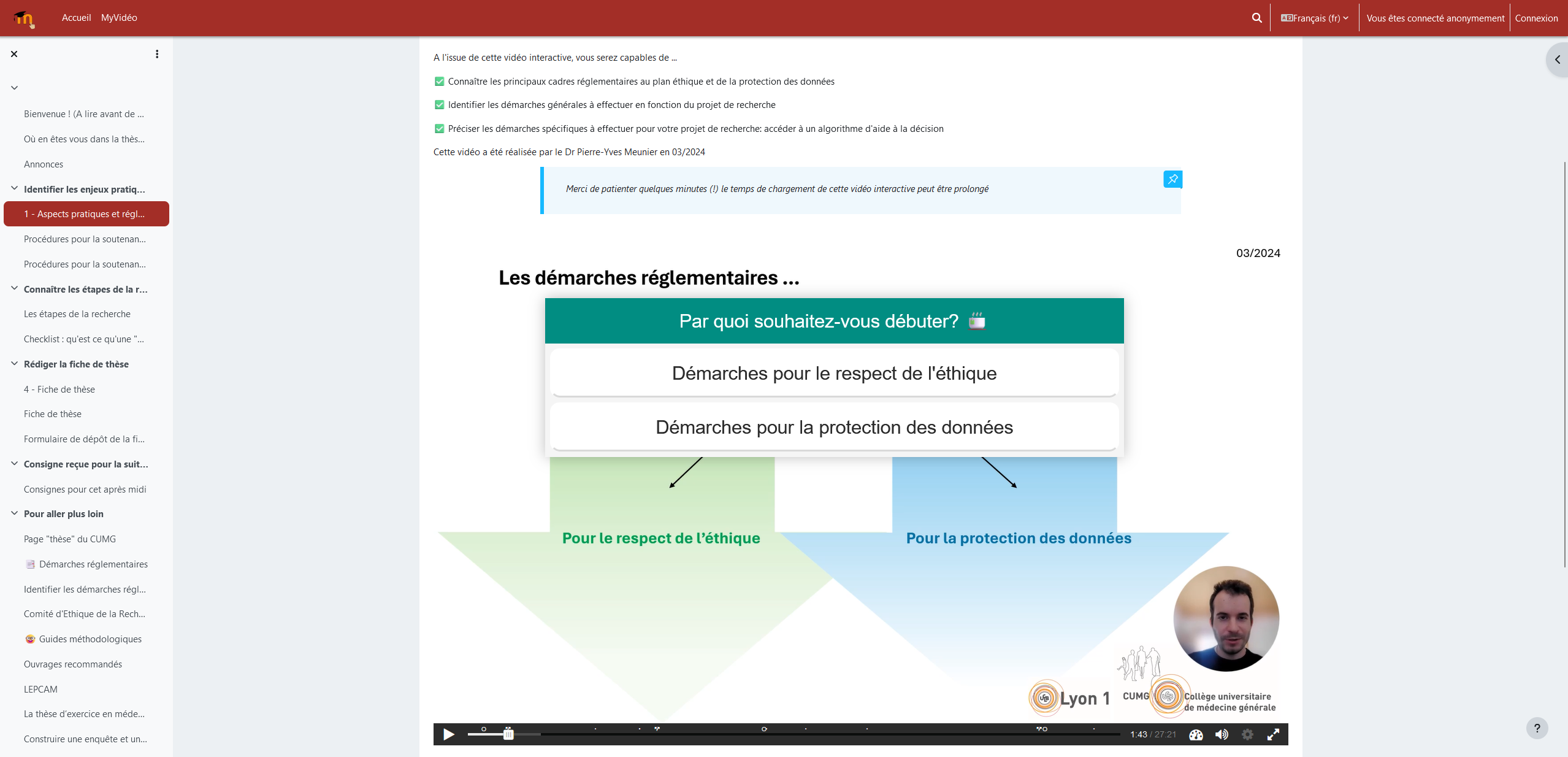
**

**
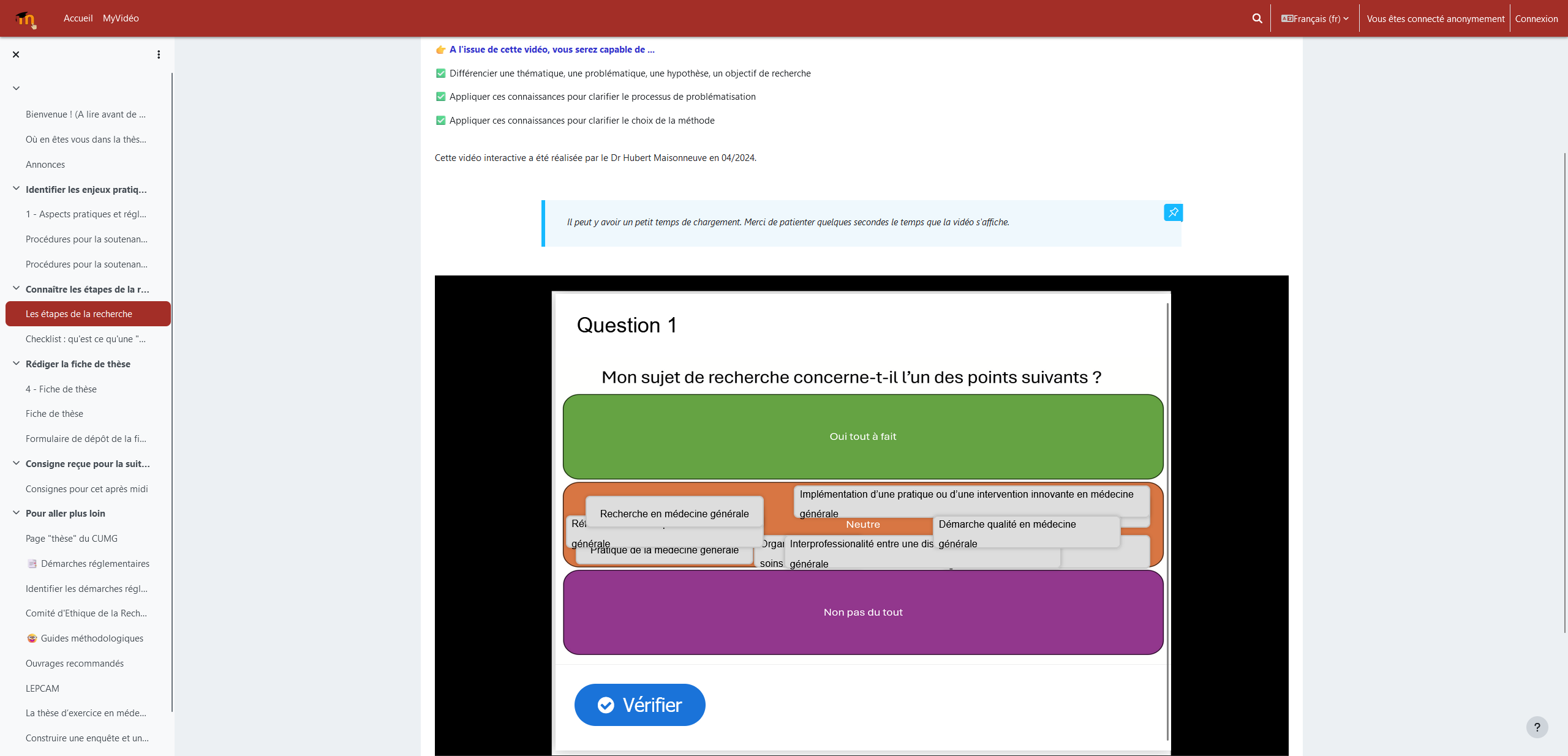
**

**
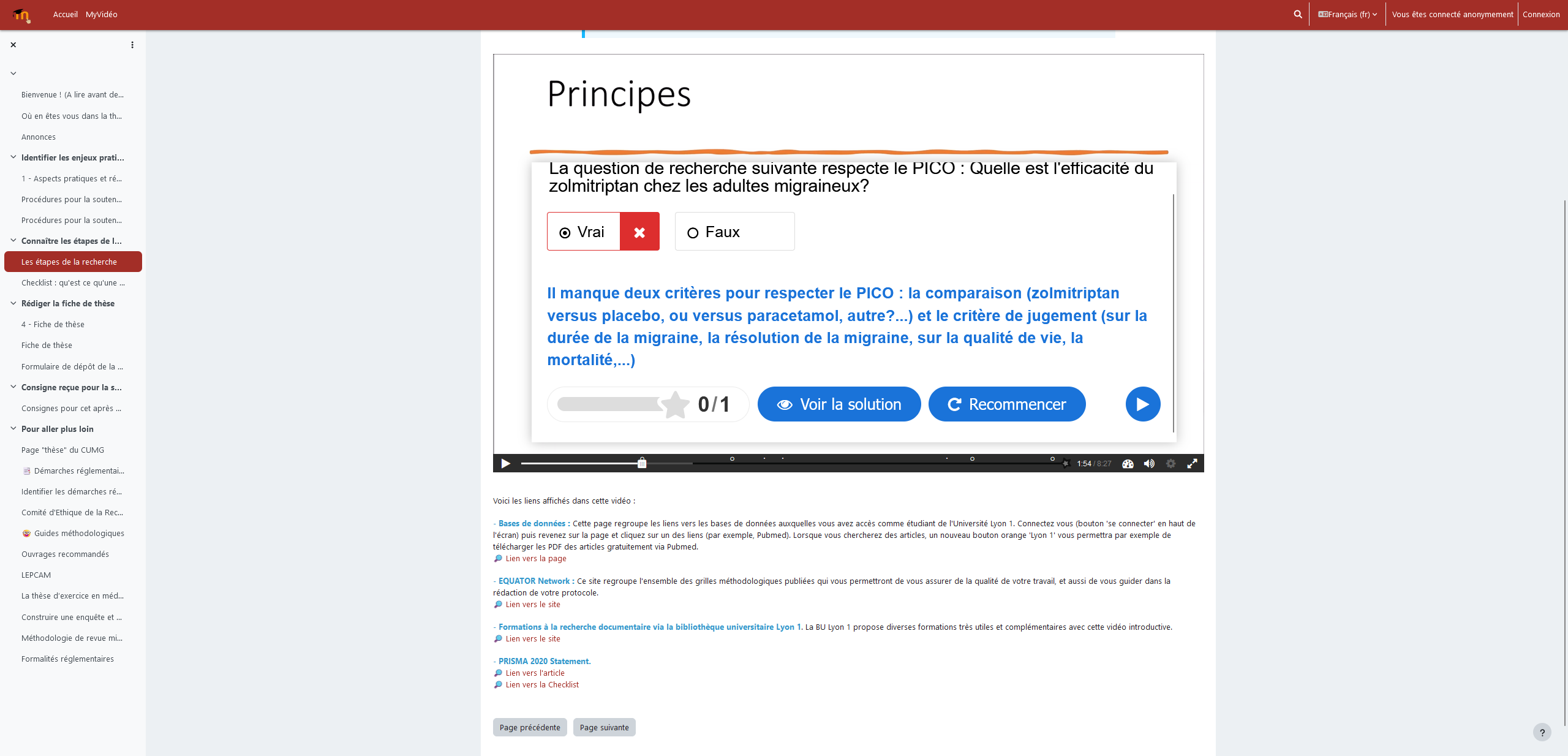
**

**
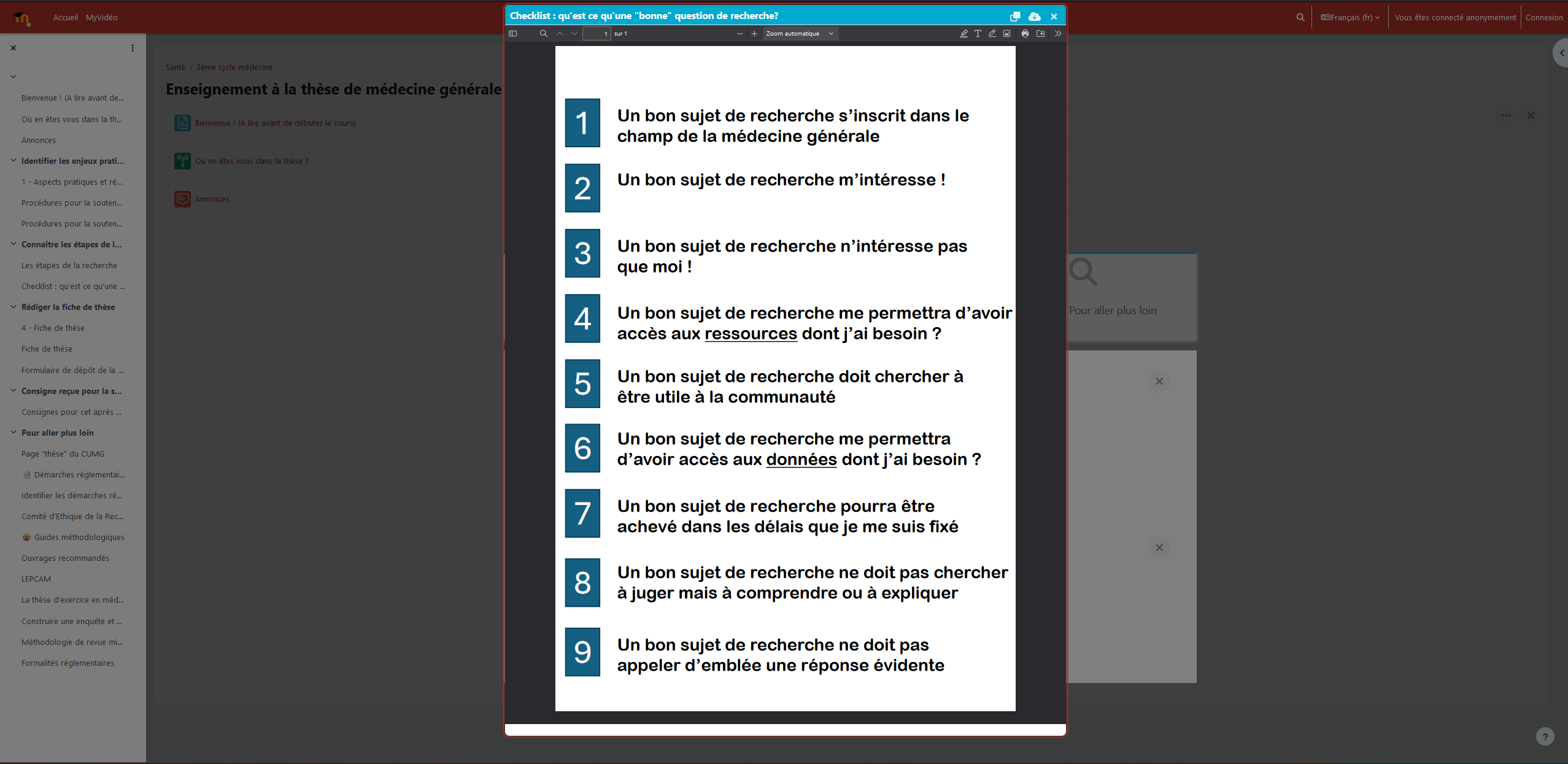


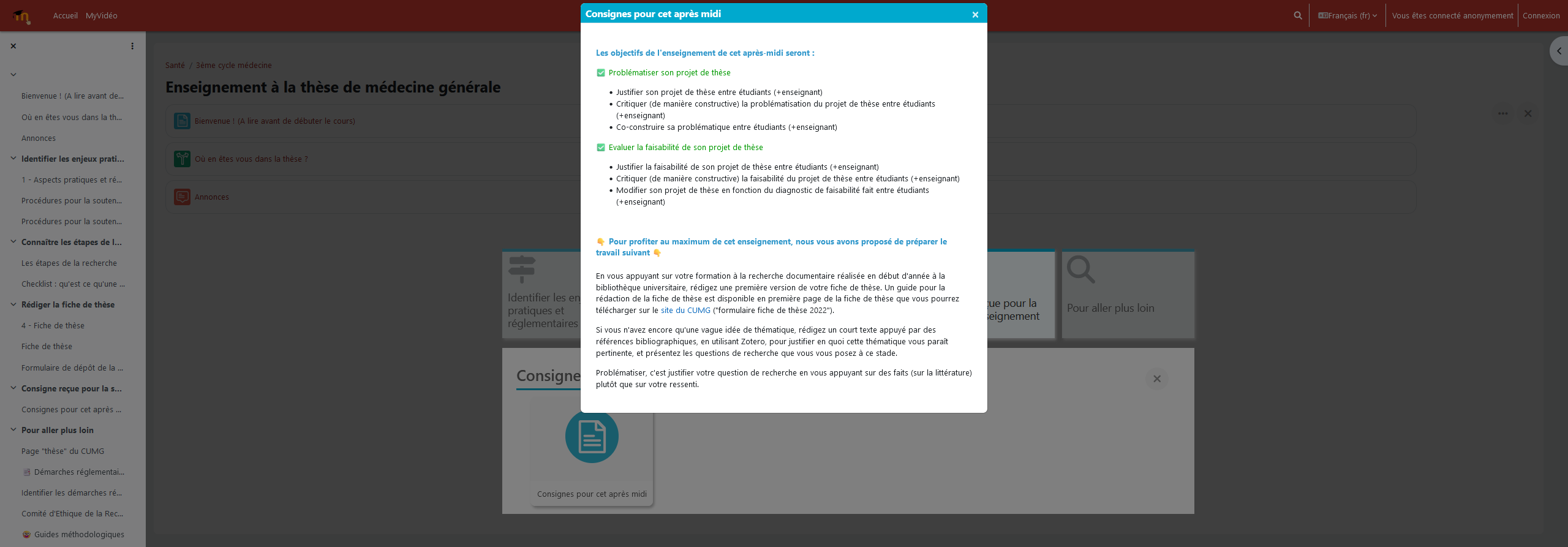
**

**
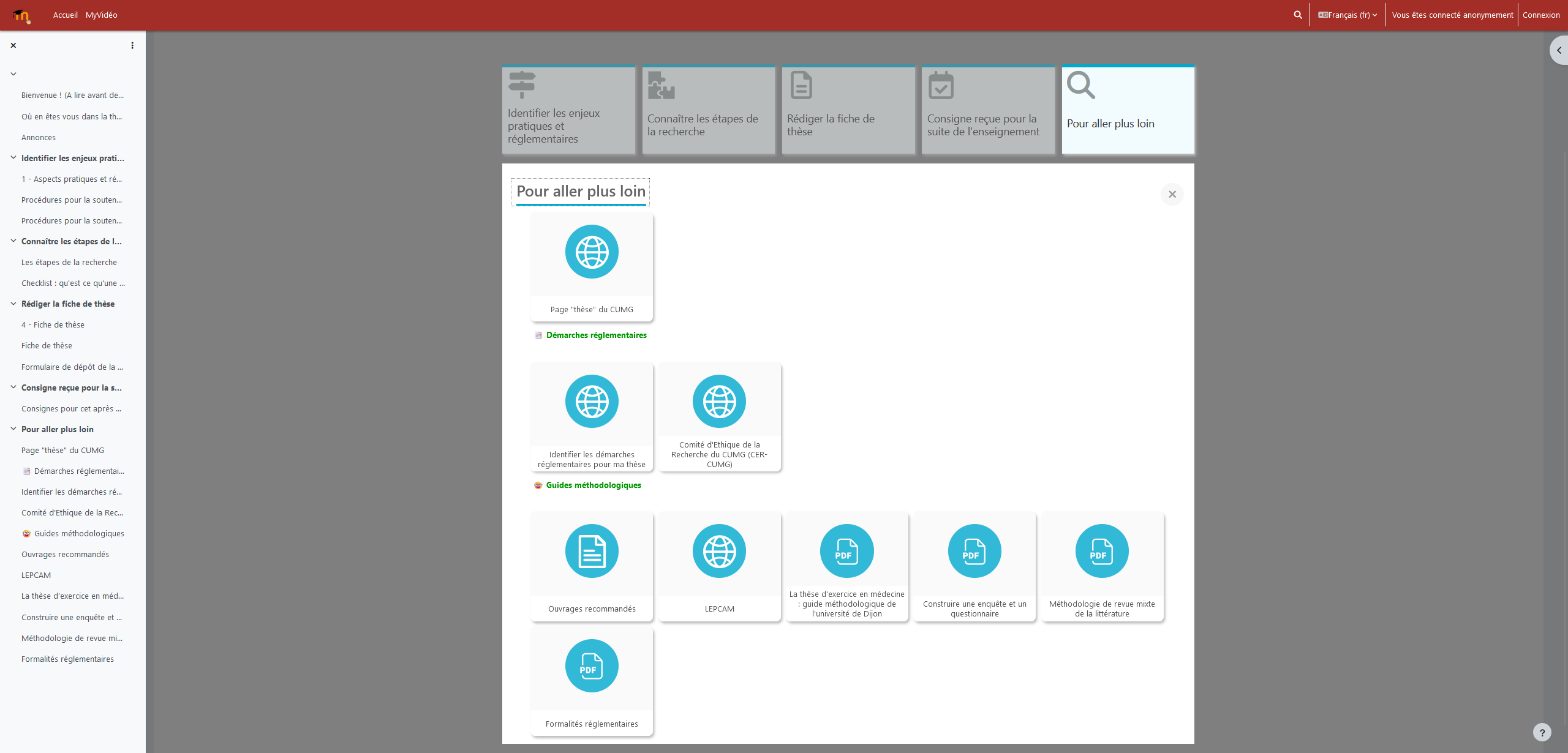
**

Supplement: Multimedia Appendix 1 [file mededu-v12-e86387-s001.docx]

**Multimedia Appendix 2. Data collection form**


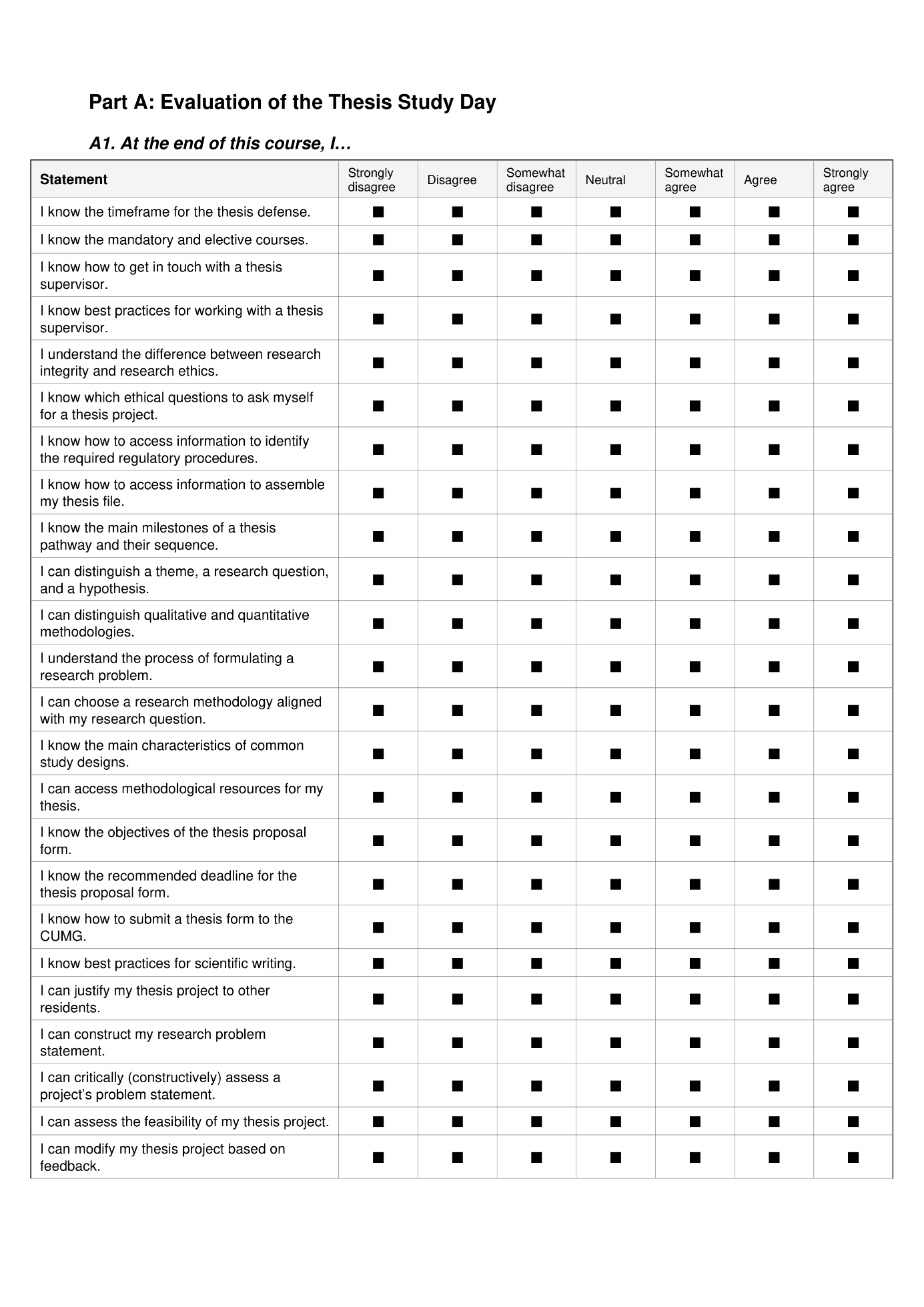

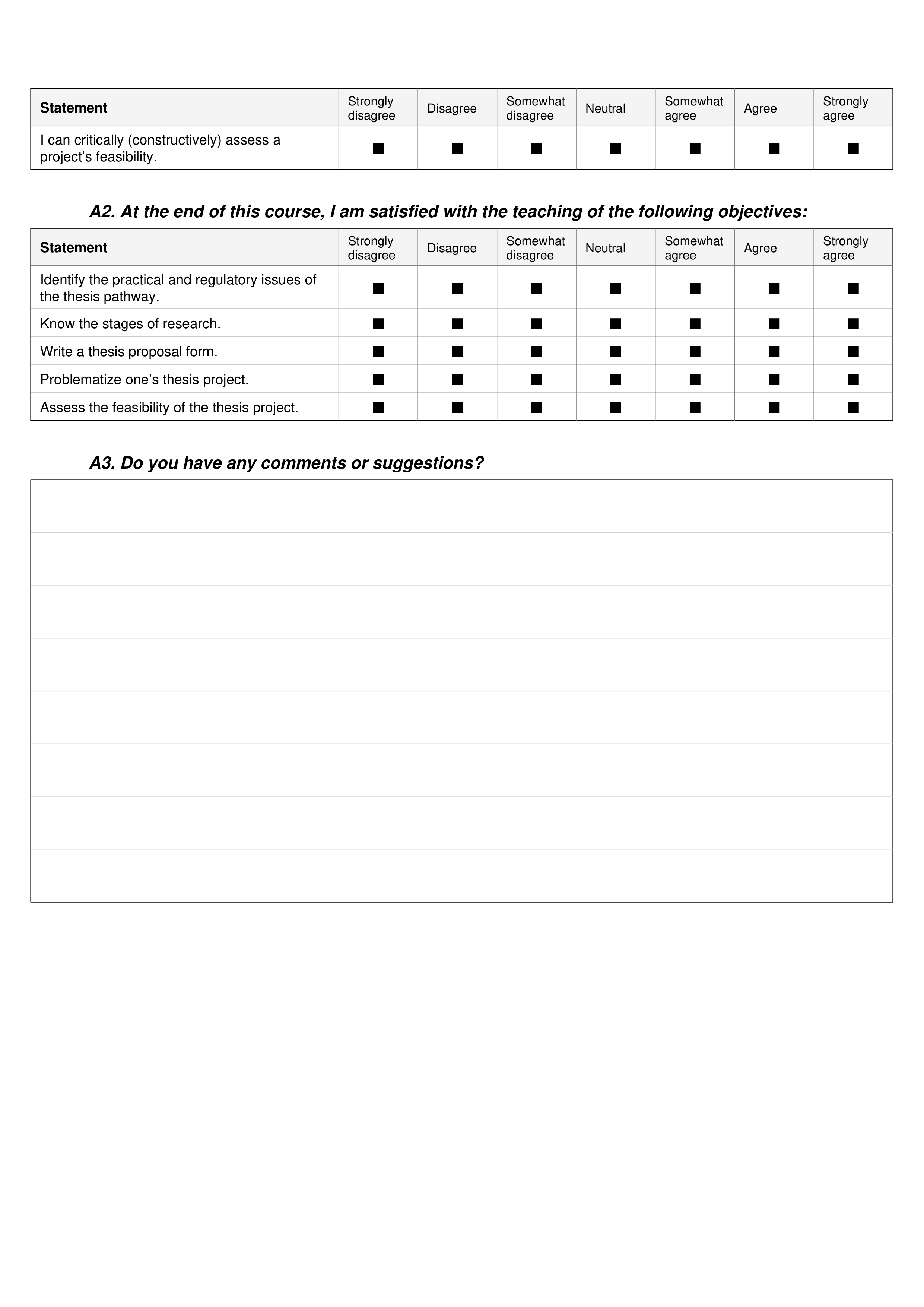

Supplement: Multimedia Appendix 2 [file mededu-v12-e86387-s002.docx]
