# Supplementary material for: Blended Learning Compared With Face-to-Face Learning Among Family Medicine Residents: Randomized Controlled Trial
Source: JMIR Med Educ. 2026 Feb 4;12:e86387. doi: 10.2196/86387 (PMC12871943; doi:10.2196/86387)
Supplement: Multimedia Appendix 3 [file mededu-v12-e86387-s003.docx]

**Multimedia Appendix 3. Questionnaire items for self-assessment and satisfaction (verbatim wording from intended learning outcomes)**


PO: primary outcome; SO: secondary outcome
