# Supplementary material for: Blended Learning Compared With Face-to-Face Learning Among Family Medicine Residents: Randomized Controlled Trial
Source: JMIR Med Educ. 2026 Feb 4;12:e86387. doi: 10.2196/86387 (PMC12871943; doi:10.2196/86387)
Supplement: Multimedia Appendix 4 [file mededu-v12-e86387-s004.docx]

**Multimedia Appendix 4:** **Multiple linear regression model: checking assumptions**


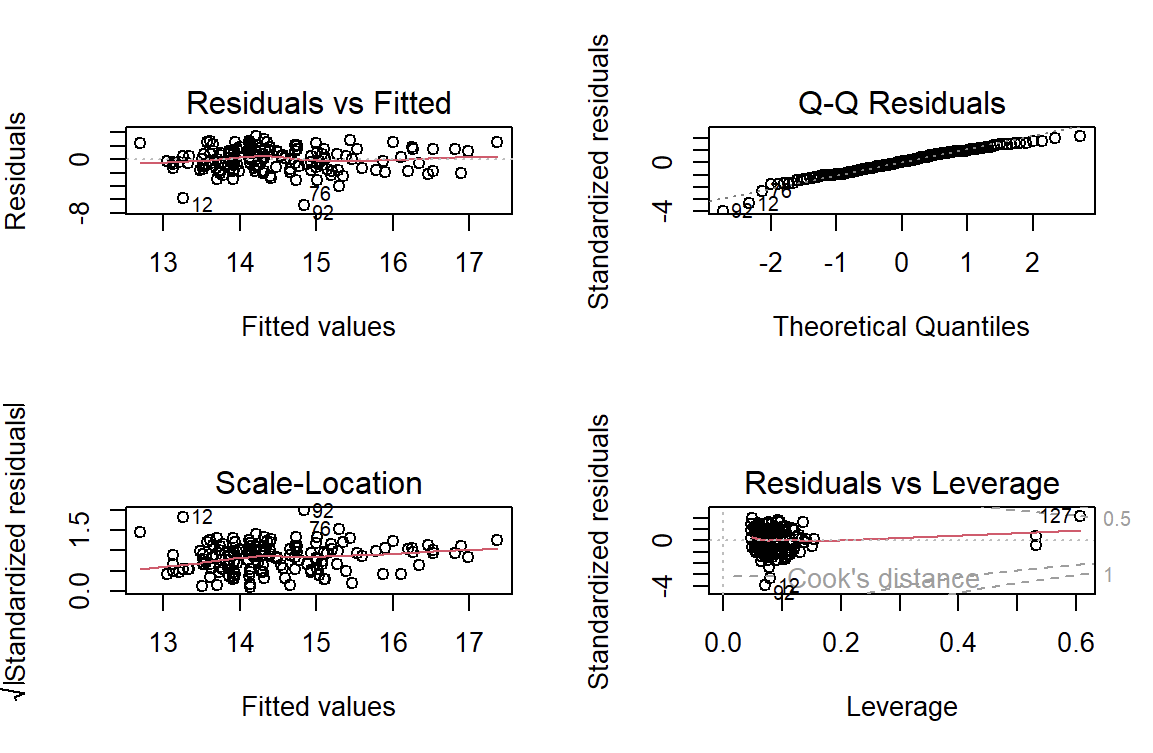


1. **Linearity**: The relationship between the independent variables and the dependent variable is linear (see Residuals vs. Fitted graph).
2. **Independence of residuals**: The residuals are independent.
   Durbin–Watson test: DW = 2.03, p-value = 0.46
3. **Homoscedasticity**: The residuals have constant variance (see Scale-Location graph).
   Breusch–Pagan test: BP = 11.33, df = 13, p-value = 0.58
4. **Normality of residuals**: Three statistical outliers contribute to a *p*-value < 0.05 in the Shapiro–Wilk test (see Q-Q plot of residuals). Indeed, the Shapiro–Wilk test computed from the data without outliers shows the normality of the residuals. Complementary analyses using the Anderson–Darling and Cramer–von Mises tests are reassuring.

   Shapiro–Wilk: W = 0.97, p-value = 0.002
   Anderson–Darling: A = 0.55, p-value = 0.16

Cramer–von Mises: W = 0.06 p-value = 0.36

A second multiple linear regression model, excluding outliers, showed no differences in the results. This is also supported by the Cook’s distance of less than 1 for the three statistical outliers (see Residuals vs. Leverage graph), indicating that these points do not have a disproportionate influence on the model.

The model appears to have no significant issue with multicollinearity, as indicated by the GVIF values:

| **Predictor variable** | **GVIF** | **Df** |
| --- | --- | --- |
| Research problem framed before the course | 1.05 | 1 |
| Research experience | 1.15 | 1 |
| Unblinding | 1.22 | 1 |
| Teachers | 2.76 | 4 |
| Another e-learning course completed on Moodle | 1.07 | 1 |
| Age | 1.09 | 1 |
| Gender | 1.13 | 2 |
| Course day (day 2) | 1.70 | 1 |
| Arm (traditional course) | 1.78 | 1 |
