# Supplementary material for: Blended Learning Compared With Face-to-Face Learning Among Family Medicine Residents: Randomized Controlled Trial
Source: JMIR Med Educ. 2026 Feb 4;12:e86387. doi: 10.2196/86387 (PMC12871943; doi:10.2196/86387)
Supplement: Multimedia Appendix 5 [file mededu-v12-e86387-s005.docx]

**Multimedia Appendix 5**: Reasons for post randomization exclusion in complete-case analysis

The only criterium for post randomization exclusion was missing data. There was no deviation from study protocol.

Eight participants (4.9%) did not provide end-of-day outcome data : n = 6/84 (7.1%) in the hybrid course arm and n = 2 (2.5%) in the traditional course arm.

In 5 cases (3 in hybrid course arm and 2 in traditional course arm), participants left the class early for work-related reasons (e.g., scheduled hospital duty outside the city or the need to arrive on time for a night shift).

Three participants (all in the blended course arm) did not complete the questionnaire at the end of the day despite being present in the classroom. They were in the same class with the same teacher. This was attributed to insufficient emphasis on the importance of completing the questionnaire.

Fisher’s exact test showed no evidence of a difference in attrition between the two allocation groups (p = 0.28).
