# Supplementary material for: Blended Learning Compared With Face-to-Face Learning Among Family Medicine Residents: Randomized Controlled Trial
Source: JMIR Med Educ. 2026 Feb 4;12:e86387. doi: 10.2196/86387 (PMC12871943; doi:10.2196/86387)
Supplement: Multimedia Appendix 6 [file mededu-v12-e86387-s006.docx]

**Multimedia Appendix 6.** **Internal consistency of family medicine residents self-assessment: Cronbach’s alphas for each educational objective**

| **Educational objective** | **Knowledge or skills-oriented** | **Items (*n*)** | **Cronbach’s alpha** |
| --- | --- | --- | --- |
| Identify the practical organization and regulatory requirements of the thesis | Knowledge | 8 | 0.695 |
| Understand the different steps of a research project | Knowledge | 7 | 0.733 |
| Draft a thesis proposal form | Knowledge | 4 | 0.473 |
| Frame the research question for a thesis project | Skills | 3 | 0.706 |
| Evaluate the feasibility of a thesis project | Skills | 3 | 0.774 |
